# When Hyperthyroidism Slows the Heart: Graves’ Disease Complicated by a High-Grade Atrioventricular Block

**DOI:** 10.7759/cureus.93581

**Published:** 2025-09-30

**Authors:** Moshanti M Ramdath, Joel D Teelucksingh

**Affiliations:** 1 Internal Medicine/ Endocrinology and Diabetes, San Fernando General/Teaching Hospital, San Fernando, TTO; 2 Internal Medicine/Endocrinology and Diabetes, San Fernando General /Teaching Hospital, San Fernando, TTO

**Keywords:** atrioventricular block, beta-blockers, graves’ disease, hyperthyroidism, pacemaker, tachyarrhythmia

## Abstract

This case report describes an uncommon presentation of Graves’ disease (GD) with a high-degree atrioventricular block (AVB). We describe a previously healthy teenage male who presented with clinical and biochemical features of GD. Shortly after starting standard therapy, he experienced a syncopal episode and was found to have a tachyarrhythmia with high-degree AVB and intermittent asystole. Cardiopulmonary resuscitation was initiated, followed by temporary pacing and eventual permanent pacemaker (PPM) implantation. Although he later stabilized, adherence to antithyroid medication remained suboptimal.

This case highlights a rare but serious presentation of GD, in which conduction system involvement necessitated both temporary and permanent pacing. Management was further complicated by the use of beta-blockers, the challenge of achieving euthyroid status, and the absence of clear guidelines for pacing in this context.

Clinicians should maintain a high index of suspicion for conduction disturbances in hyperthyroid patients presenting with syncope. This case underscores the need for vigilance regarding conduction disturbances in thyrotoxic patients. It highlights the urgent need for multicenter data to guide long-term pacing strategies in this unique overlap of endocrinology and cardiology.

## Introduction

Graves’ disease (GD), an autoimmune condition first described by Robert Graves in 1835, accounts for the majority of hyperthyroidism cases globally [1]. While cardiovascular manifestations such as atrial fibrillation, sinus tachycardia, and supraventricular tachycardias are well documented, bradyarrhythmias and higher-degree atrioventricular blocks (AVBs) are exceedingly rare, with less than 35 reported cases involving both adult and pediatric populations [2-4]. Reports of AVB in hyperthyroidism are typically associated with secondary triggers such as infection, electrolyte imbalances, medications, or systemic inflammation [3]. High-degree AVBs are life-threatening since progression to complete heart block can occur. Hence, prompt identification and management are required with possible pacing for improved outcomes. In this report, we describe a case of symptomatic high-degree AVB in a teenage male with GD, with no other identifiable cause, managed with temporary pacing and eventual permanent pacemaker (PPM) insertion.

## Case presentation

A previously healthy teenage male presented with a three-month history of weight loss (9 kg), heat intolerance, palpitations, tremor, and fatigue. There was no history of any recent immunizations, travel, infections, or sick contacts. He denied consumption of any herbal medications or supplements but smoked cannabis occasionally. Physical examination revealed a goitrous thyroid with bruit, mild exophthalmos, and fine tremors. Vital signs showed a pulse of 104 beats per minute (bpm) and blood pressure of 129/74 mmHg. A baseline electrocardiogram showed sinus tachycardia. The biochemistry panel and complete blood count were within normal range. A chest X-ray revealed no consolidation, effusions, or masses with a normal cardio-thoracic ratio.

Thyroid ultrasound scan revealed a heterogeneous, hypervascular, and enlarged appearance throughout with no focal nodules or cystic collections. The isthmus measured 0.97 cm, the right lobe measured 8.2 cm x 4.0 cm x 4.2 cm, and the left lobe measured 9.9 cm x 3.9 cm x 4.1 cm. There was no evidence of cervical lymphadenopathy or retrosternal extension. Laboratory investigations confirmed hyperthyroidism secondary to GD (Table 1). The Burch-Wartofsky score was 15 (low risk for thyroid storm).

**Table 1:** Summary of laboratory investigation results Values in bold indicate significant findings.

TEST (SERUM)	RESULT	REFERENCE RANGE
Thyroid-stimulating hormone	0.01 IU/ml	0.27-4.2 IU/ml
Free thyroxine	24.9 ng/dl	5.1-14.1 ng/dl
Free triiodothyronine	6.5 ng/dl	0.8-1.3 ng/dl
Thyrotropin receptor antibody	14.4 IU/L	< 1.5 IU/L

**Figure 1:**
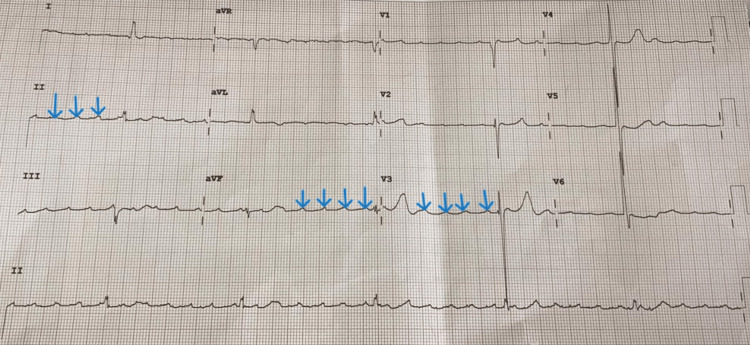
A 12-lead electrocardiogram, obtained during syncopal episode, showing a high-degree atrioventricular block; Mobitz type 2 (3:1 and 4:1 conduction ratio)

**Figure 2:**
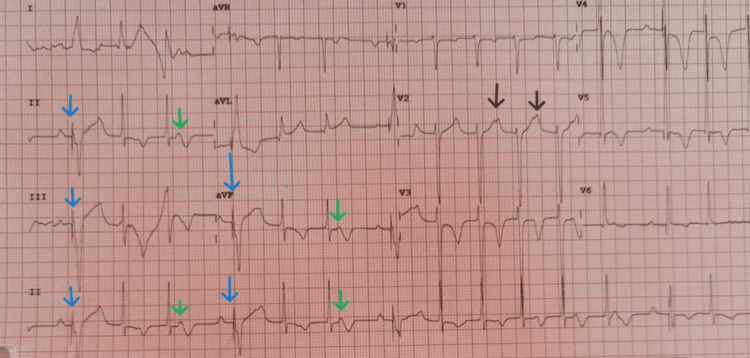
A 12-lead electrocardiogram (post temporary transvenous pacing) showing paced complexes (blue arrow), a first-degree atrioventricular block (green arrow), and a second-degree atrioventricular block with a 2:1 conduction ratio (black arrow)

He was initiated on carbimazole and propranolol. On Day 1 post admission, he suffered a syncopal episode and was found unresponsive with a Glasgow Coma Scale (GCS) score of 3/15. Monitoring revealed a fast atrial rhythm with ventricular rates <20 bpm and intermittent asystole (Figure 1). Cardiopulmonary resuscitation was initiated, propranolol was discontinued, and external pacing commenced immediately. The patient stabilized; however, because of intermittent episodes of atrial tachycardia with high-degree AVB on follow-up electrocardiograms (Figure 2), the decision for dual-chamber PPM insertion was undertaken (four days post temporary pacing). Of note, his echocardiogram was structurally normal, and there were no signs of infection, ischemia, or metabolic derangements. A brain CT was also normal, with no gross intracranial pathology noted.

**Figure 3:**
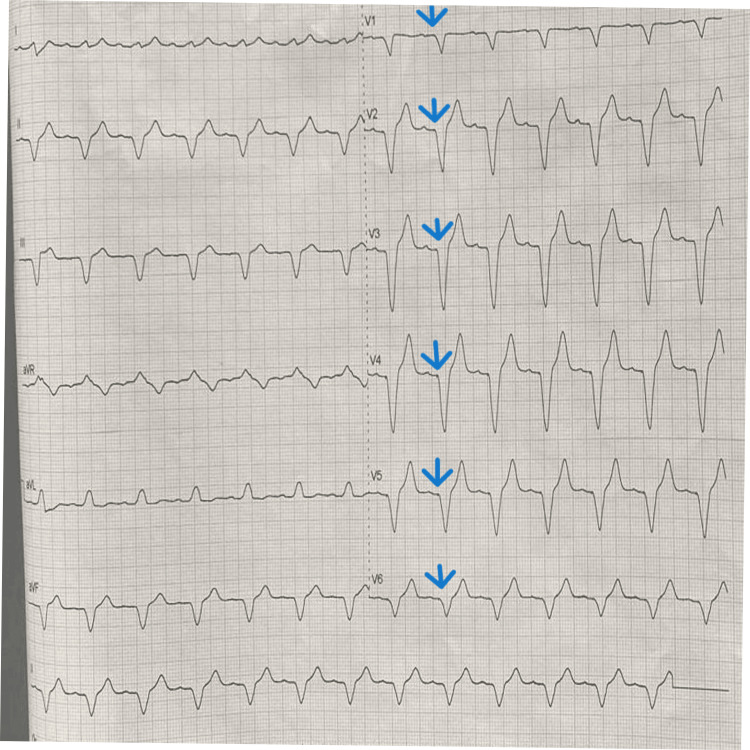
A 12-lead electrocardiogram showing wide QRS complexes with pacemaker spikes as indicated by arrows, preceded by a corresponding p wave indicating atrial-sensed and ventricular-paced complexes post permanent pacemaker (PPM) implantation.

Following PPM insertion, the patient stabilized clinically (Figure 3). However, he remained poorly compliant with both endocrine and cardiac clinic follow-up as well as antithyroid medication. His first presentation to the cardiac clinic occurred at 10 months post PPM implantation. He remained asymptomatic with an intrinsic rhythm in >99% of pacing checks. Despite the occasional cannabis use, emphasis on cessation was reinforced. He was subsequently counseled regarding pacemaker removal. His thyroid function remained uncontrolled due to non-adherence to antithyroid therapy, with ongoing discussions about definitive treatment options.

## Discussion

Although cardiovascular manifestations of thyrotoxicosis are common, the development of high-degree AVB is rare and often perplexing [3, 5, 6]. Early case reports dating back to the Mayo Clinic in 1933 described this phenomenon predominantly in older adults and women, often with concurrent infections [3]. In contrast, our patient was a healthy young male with no identifiable secondary causes other than occasional use of cannabis. 

The pathophysiology remains speculative, ranging from autoimmune myocarditis leading to infiltration of cardiac fibers and direct thyrotoxic effect on the conduction system to increased vagal tone [3]. Cannabis use is reported to cause AVB via alterations in the autonomic nervous system, leading to enhanced parasympathetic stimulation [7]. Treatment strategies vary, with many cases resolving following the achievement of euthyroid status. However, symptomatic patients with hemodynamic instability like ours may require temporary and even permanent pacing. Both Ozcan et al. and Kuo et al. reported cases in the literature whereby symptomatic hyperthyroid patients with AVB were managed with permanent PPM coupled with antithyroid medication [6, 8]. Our patient, after temporary pacing, also had intermittent episodes of atrial tachycardia and high-degree AVB, thus supporting the role for PPM insertion. Propranolol, though standard in GD, can unmask latent conduction disease; hence the importance of obtaining a baseline electrocardiogram prior to commencing treatment with this drug [3,5]. Following the syncopal episode and the diagnosis of high-degree AVB in our patient, the propranolol was discontinued.

Current European Society of Cardiology/European Heart Rhythm Association guidelines recommend pacing in the absence of a reversible cause only if patients are symptomatic or if there is a high risk of progression to complete heart block [9]. These guidelines are similar for both pediatric and adult cases. However, data remain limited when thyrotoxicosis is the underlying etiology [3,9]. Furthermore, the optimal timing for PPM removal in reversible cases remains unclear [6]. This case raises the clinical dilemma: Should all high-degree AVBs in hyperthyroid patients receive temporary pacing only, or does a subset require early permanent pacing?

## Conclusions

This case illustrates the rare but life-threatening association of GD with high-degree AVB. Clinicians should maintain a high index of suspicion in hyperthyroid patients presenting with syncope, as prompt recognition and pacing can be lifesaving. In our patient, progression to permanent pacing occurred within four days following temporary pacing due to intermittent episodes of atrial tachycardia with high-degree AVB. However, in the presence of a reversible cause such as thyrotoxicosis, pacing in this context remains uncertain. Prompt intervention in hemodynamically unstable patients, however, appears justified. Future studies linking GD to conduction system pathologies can be beneficial in addition to larger registries and collaborative data to guide long-term pacing strategies in this overlap between endocrine and cardiac disease.
